# Using hydrogen isotopes of freshwater fish tissue as a tracer of provenance

**DOI:** 10.1002/ece3.2519

**Published:** 2016-10-05

**Authors:** David X. Soto, Keith A. Hobson, Leonard I. Wassenaar

**Affiliations:** ^1^ Environment Canada Saskatoon SK Canada; ^2^ Present address: International Atomic Energy Agency Vienna Austria

**Keywords:** aquatic organisms, deuterium, fish, food webs, Lake Winnipeg, size effects, stable isotopes

## Abstract

Hydrogen isotope (δ^2^H) measurements of consumer tissues in aquatic food webs are useful tracers of diet and provenance and may be combined with δ^13^C and δ^15^N analyses to evaluate complex trophic relationships in aquatic systems. However, δ^2^H measurements of organic tissues are complicated by analytical issues (e.g., H exchangeability, lack of matrix‐equivalent calibration standards, and lipid effects) and physiological mechanisms, such as H isotopic exchange with ambient water during protein synthesis and the influence of metabolic water. In this study, δ^2^H (and δ^15^N) values were obtained from fish muscle samples from Lake Winnipeg, Canada, 2007–2010, and were assessed for the effects of species, feeding habits, and ambient water δ^2^H values. After lipid removal, we used comparative equilibration to calibrate muscle δ^2^H values to nonexchangeable δ^2^H equivalents and controlled for H isotopic exchange between sample and laboratory ambient water vapor. We then examined the data for evidence of trophic δ^2^H enrichment by comparing δ^2^H values with δ^15^N values. Our results showed a significant logarithmic correlation between fork length and δ^2^H values, and no strong relationships between δ^15^N and δ^2^H. This suggests the so‐called apparent trophic compounding effect and the influence of metabolic water into tissue H were the potential mechanisms for δ^2^H enrichment. We evaluated the importance of water in controlling δ^2^H values of fish tissues and, consequently, the potential of H isotopes as a tracer of provenance by taking account of confounding variables such as body size and trophic effects. The δ^2^H values of fish appear to be a good tracer for tracking provenance, and we present a protocol for the use of H isotopes in aquatic ecosystems, which should be applicable to a broad range of marine and freshwater fish species. We advise assessing size effects or working with fish of relatively similar mass when inferring fish movements using δ^2^H measurements.

## 1 Introduction

The hydrogen isotope (δ^2^H) composition of animal tissues has been successfully used to decipher animal migration patterns in terrestrial ecosystems (Hobson & Wassenaar, 2008) and recently were used to quantify the relative contribution of, and connections among, aquatic and terrestrial food webs (Babler, Pilati, & Vanni, 2011; Voigt, Lehmann, & Greif, 2015). Tracking terrestrial animal movements with H isotopes is based on the linkage of spatial correlations of deuterium in precipitation (Bowen, Wassenaar, & Hobson, 2005; Terzer, Wassenaar, Araguás‐Araguás, & Aggarwal, 2013) with those found in animal tissues. Recent applications using δ^2^H measurements have shown potential to provide insights to trace energy flows and trophic patterns in aquatic food web studies (reviewed in Vander Zanden, Soto, Bowen, & Hobson, 2016). An evaluation of the behavior of hydrogen isotopes of biota in aquatic ecosystems is important because of its link to environmental water isotopic patterns, and potential to answer questions intractable using C or N isotopes linked mainly to diet. Nonetheless, δ^2^H measurements are complicated by multiple hydrogen sources (diet and water), and some have emphasized the need for controlled studies to investigate this complexity (Jardine, Kidd, & Cunjak, 2009; Podlesak et al., 2008) and field‐based studies to better prove the application for tracking provenance of species in aquatic systems.

Experimental data show that in aquatic systems, H isotopes are tracers of diet and provenance (Solomon et al., 2009; Soto, Wassenaar, & Hobson, 2013c), with potential to link consumer δ^2^H values to dietary sources (Cole & Solomon, 2012) and environmental water (Whitledge, Johnson, & Martinez, 2006). The use of δ^2^H as a dietary source tracer in aquatic systems is based on the fact that the H isotopic composition of terrestrial vs. aquatic sources can differ significantly (Doucett, Marks, Blinn, Caron, & Hungate, 2007), in contrast to δ^13^C and δ^15^N measurements, which have little resolving power in many aquatic systems (Fry, 2013; Soto, Gacia, & Catalan, 2013a). In aquatic organisms, increases in consumer δ^2^H values with trophic level are related to the “compounding effect” of H isotope exchange between ambient water and tissue formed at each trophic step. Newly synthesized tissue incorporates H atoms from environmental water (as well as from diet) at each trophic step. Generally, water H isotopic composition is relatively enriched in deuterium compared to food web components, and the contribution of water H into animal tissues thus creates a progressive enrichment in δ^2^H along food chains. The magnitude of the compounding effect depends largely upon the H isotopic differences between water and dietary H (Solomon et al., 2009; Soto et al., 2013c). This process does not seem to discriminate the isotopic composition derived from diet H during food assimilation and digestion.

To date, the use of δ^2^H measurements in aquatic ecology was limited due to analytical issues (Wassenaar, Hobson, & Sisti, 2015) and the unknown contribution to tissues of water derived from the environment and metabolism (Soto, Hobson, & Wassenaar, 2013b; Soto et al., 2013c). Analytical issues include uncontrolled H isotopic exchange between sample and ambient water vapor and the presence of other confounding effects such as the presence of δ^2^H‐depleted lipids. There remains inconsistency among published analytical methods—some authors normalize H isotope values using matrix‐equivalent laboratory working standards with similar exchangeable H properties, and remove lipids prior to δ^2^H analysis, while others do not (see review in Meier‐Augenstein, Hobson, & Wassenaar, 2013). The proportion of tissue H derived from ambient water in each trophic step needs to be estimated or assumed, and to date, studies show considerable variability among aquatic organisms (between 10%–50%; Solomon et al., 2009; Wang, O'Brien, Jenson, Francis, & Wooller, 2009; Nielson & Bowen, 2010; Soto et al., 2013c). It remains unknown whether the water fraction of H is the cause of this variability or is related to other effects such as metabolism or incomparable analytical methods used. Preliminary findings have demonstrated the effect of fish body size on δ^2^H values (Soto, Wassenaar, Hobson, & Catalan, 2011), and the metabolic mechanism to explain this pattern was investigated under laboratory conditions (Soto et al., 2013b). The degree of incorporation of H derived from dietary lipids—depleted in δ^2^H—to tissue H was ultimately influenced by growth rate. Small‐sized fish with relatively higher growth rates have comparatively lower δ^2^H values than larger fish. Taking into account all these issues, the question remains whether hydrogen isotopes of fish tissue can be used as tracers of provenance.

Lake Winnipeg is the tenth largest freshwater lake in the world and has a dual‐basin hydrologic system. The distinctive catchment and nutrient inputs into the Lake Winnipeg are mainly the Red and Winnipeg Rivers in the south basin, and the Saskatchewan River on the west side of the north basin. The waters of the north basin are mixed with south basin waters that flow north through the Narrows. The outflow of the lake is located in the north basin through the Nelson River, which drains their waters into Hudson Bay. The eutrophic state of the lake is mainly due to increasing nutrient loadings in the south basin from the Red River and, to a lesser extent, from the Winnipeg River (Mayer, Simpson, Thorleifson, Lockhart, & Wilkinson, 2006; McCullough et al., 2012). Consequently, the impacts of eutrophication in the lake during the last decades have been significant, for instance, extensive algal blooms. The hydrology of Lake Winnipeg provides a unique aquatic system with a distinctive baseline isotopic structure within the lake with relevant implications for provenance and food web studies. The spatial isotopic differences of hydrogen isotope values in lake water due to catchment inputs (Zhao, Rao, & Wassenaar, 2012) provide a sufficient H water isotopic baseline to evaluate fish provenance. In addition, the basin‐specific carbon and nitrogen isotopic patterns for nutrient sources and ultimately fish communities (Hobson, Ofukany, Soto, & Wassenaar, 2012) require the north and the south basin of the lake be treated separately when using stable isotopes as food web tracers.

We conducted a field study to examine δ^2^H variability in freshwater fish tissue obtained from Lake Winnipeg, Canada (2007–2010). We compared lakewide water isotope patterns to resident fish H isotope data. We analyzed all fish tissue samples using well‐established controls for exchangeable hydrogen in organic materials, as well as removing lipids. We examined: (1) the importance of water δ^2^H in determining the H isotope composition of fish tissues and (2) assess the evidence for H isotope trophic‐enrichment effect similar to that seen for δ^15^N values in this fish community. For each objective, we considered all possible confounding factors (i.e., size, water, diet).

## 2 Material and methods

### 2.1 Sample collection

**Figure 1:**
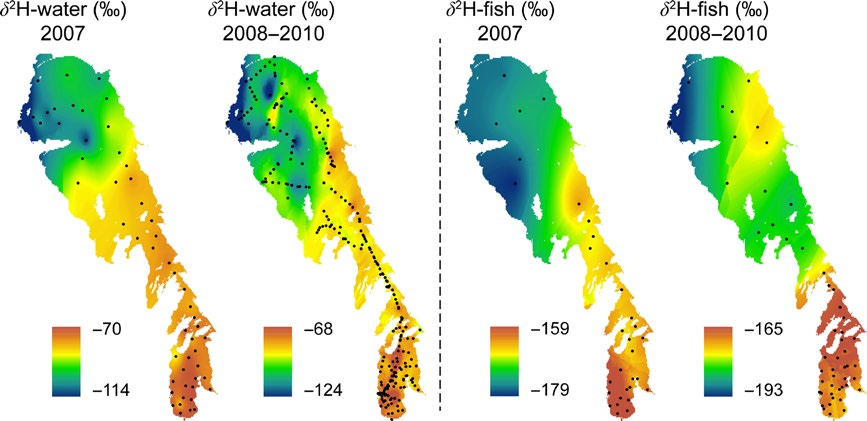
Spatial patterns in hydrogen isotope values of water and fish (<250 mm) in 2007 and the period of 2008–2010. Black dots depict the sampled stations used for the interpolated (kriging) maps for each time period

Filtered surface water samples from a well‐mixed water column were collected at stations located across Lake Winnipeg over four seasons (winter, spring, summer, and fall) of 2007–2010, except for the summer and fall of 2010 (Figure 1). Water samples were stored in tightly sealed HDPE bottles. All water isotope sampling locations, data, and spatial isotope patterns in Lake Winnipeg used in this study were taken from Beveridge et al. (2012) and Zhao et al. (2012).

Fish samples were collected from archived samples from isotope investigations obtained from trawls taken at the same stations as the water samples (Hobson et al., 2012; Ofukany, Wassenaar, Bond, & Hobson, 2014). These studies showed isotopic spatial differentiation of fish individuals across Lake Winnipeg (with δ^13^C, δ^15^N, and δ^34^S) and suggested little long‐distance fish movements within the lake. We assumed therefore that fish did not move far from the water sample collection sites. Fish were collected during spring (April–June), summer (July–August), and fall (September–November) of 2007–2010 and in winter of 2009–2010 from the south basin (Figure 1). Fish with fork lengths less than or equal to 350 mm were collected during spring, summer, and fall by trawls deployed by the *MV Namao* research vessel for 30 min at a speed of approximately 3.9 km/hr (Lumb, Franzin, & Watkinson, 2012). Commercial gill nets were also used to collect fish from larger size classes (up to 800 mm). Specimens of lake cisco (*Coregonus artedi*), emerald shiner (*Notropis atherinoides*), goldeye (*Hiodon alosoides*), mooneye (*Hiodon tergisus*), northern pike (*Esox lucius*), rainbow smelt (*Osmerus mordax*), nine‐spine stickleback (*Pungitius pungitius*), trout‐perch (*Percopsis omiscomaycus*), walleye (*Sander vitreus*), white bass (*Morone chrysops*), white sucker (*Catostomus commersonii*), and yellow perch (*Perca flavescens*) were obtained up to 350‐mm fork length. Larger specimens (>350 mm) of northern pike, walleye, and white sucker were obtained using gill nets during the winter of 2009–2010 and the ice‐free season of 2010 in the south basin.

Fish were sorted by species, and were stored frozen (−20°C), whole for small fish and filleted for larger fish, before processing at the stable isotope laboratory of the National Hydrology Research Centre in Saskatoon, Saskatchewan. Small, whole specimens were partially thawed, and dorsal muscle was excised for stable isotope analysis. All muscle samples were freeze‐dried.

### 2.2 Stable isotope measurements

Water samples were analyzed for δ^2^H/^1^H ratios using laser absorption spectroscopy (Lis, Wassenaar, & Hendry, 2008; Wassenaar, Coplen, & Aggarwal, 2013) with an off‐axis integrated cavity output spectroscopy water isotope analyzer (Los Gatos model DLT‐100) coupled to a CTC‐PAL liquid autosampler. Water isotope data were normalized to the VSMOW‐SLAP scale using two well‐calibrated laboratory water standards (summarized in Beveridge et al., 2012).

For fish muscle samples, hydrogen isotope measurements were conducted on lipid‐extracted subsamples in order to eliminate the confounding contribution of δ^2^H‐depleted lipids (Jardine et al., 2009; Soto et al., 2013c; Wilkinson, Cole, & Pace, 2015). Lipids were removed from muscle tissue using a solution of 2:1 chloroform: methanol, and samples were then allowed to air‐dry. Subsamples (0.35 ± 0.02 mg) from lipid‐extracted muscle were weighed in silver capsules and analyzed for δ^2^H measurements using pyrolysis combustion (1,350°C) and gas separation (Eurovector HT‐PyrOH, Milan, Italy) prior to continuous‐flow isotope ratio mass spectrometry (http://www.isoprime.co.uk). To control for H isotopic exchange of H atoms in tissue with ambient vapor, we used the comparative equilibration method to determine the nonexchangeable δ^2^H (Wassenaar & Hobson, 2003) using three calibrated, lipid‐free, laboratory fish muscle standards (Florida garr, FLG, −64 ‰; Atlantic salmon, ATS, −113 ‰; lake trout, LAT, −166 ‰). The isotope δ values are reported in parts per thousand (‰) deviations from the international standard (VSMOW) on the VSMOW‐SLAP scale. Within‐run analytical precisions for δ^2^H determined from repeated analyses of control standards (*n *=* *5) were better than 1.5 ‰ for water and tissue samples.

### 2.3 Statistical analyses

General linear models (GLM) were used to determine which factors influenced and drove the variability of Lake Winnipeg water isotope data. We modeled the effect of year (2007–2010), basin (north, narrows, south), and season (winter, spring, summer, fall) on water δ^2^H values. Spatial patterns in δ^2^H values in water and fish were projected using interpolation techniques (i.e., kriging) in ArcGIS for the time period of interest depending on the previous analysis obtained from the GLM models. Lastly, we investigated the relationships between muscle δ^2^H and fork length and also for potential trophic effects using δ^15^N values. For the assessment of trophic effects, we evaluated the relationship between muscle δ^2^H and δ^15^N values (Cabana & Rasmussen, 1994; Minagawa & Wada, 1984). We used bulk (non‐lipid‐extracted) tissue δ^15^N values from Hobson et al. (2012) and Ofukany et al. (2014) from the same subsamples used for δ^2^H analysis in this study.

## 3 Results

Water isotope δ^2^H data ranged from −124 ‰ to −60 ‰, and the effect of basin was the predominant factor with both basins significantly different owing to isotopically distinctive inputs and mixing in Lake Winnipeg from three contributing watersheds (Table 1, ANOVA, *F *=* *155.72; Tukey's test, *p *<* *.01). This large H isotopic range and mixing patterns offered us a potential to test and evaluate the use of hydrogen isotopes as a tracer of provenance for fish species. In relation to the other factors (Table 1), we found a significant effect between 2007 and all other years, with an average difference of ca. 5–6 ‰ (ANOVA, *F *=* *51.92; Tukey's test, *p *<* *.001) and between fall and the other seasons, with an average difference of ca. 2–3 ‰ (ANOVA, *F *=* *14.08; Tukey's test, *p *<* *.05). Throughout the article, we therefore make the distinction between 2007 and the 2008–2010 data. The differences in water isotope composition among seasons were close to the analytical precision (±2 ‰) owing to multiyear lake water residence times, and therefore, season was considered irrelevant for fish tissue δ^2^H values. Interpolated maps of water δ^2^H values showed water from the south basin was enriched in δ^2^H compared to the north basin for all periods (2007 and 2008–2010, Figure 1) along a strong longitudinal and latitudinal mixing gradient (Fig. S1).

**Table 1:** Mean ± *SD* (*n*) δ^2^H values for the water in north and south basins and narrows of Lake Winnipeg (2007–2010). Isotopic data were grouped in 2007 and the 2008–2010 data (see 3). No data were available for summer and fall 2010

Basin	Period	δ^2^H (‰)		
		*N*	Mean ± *SD*	Range
North	2007	133	−86 ± 9	[−121, −73]
	2008–2010	475	−89 ± 7	[−124, −76]
Narrows	2007	27	−77 ± 3	[−85, −72]
	2008–2010	91	−85 ± 5	[−100, −76]
South	2007	104	−75 ± 5	[−94, −62]
	2008–2010	375	−83 ± 6	[−102, −60]

**Figure 2:**
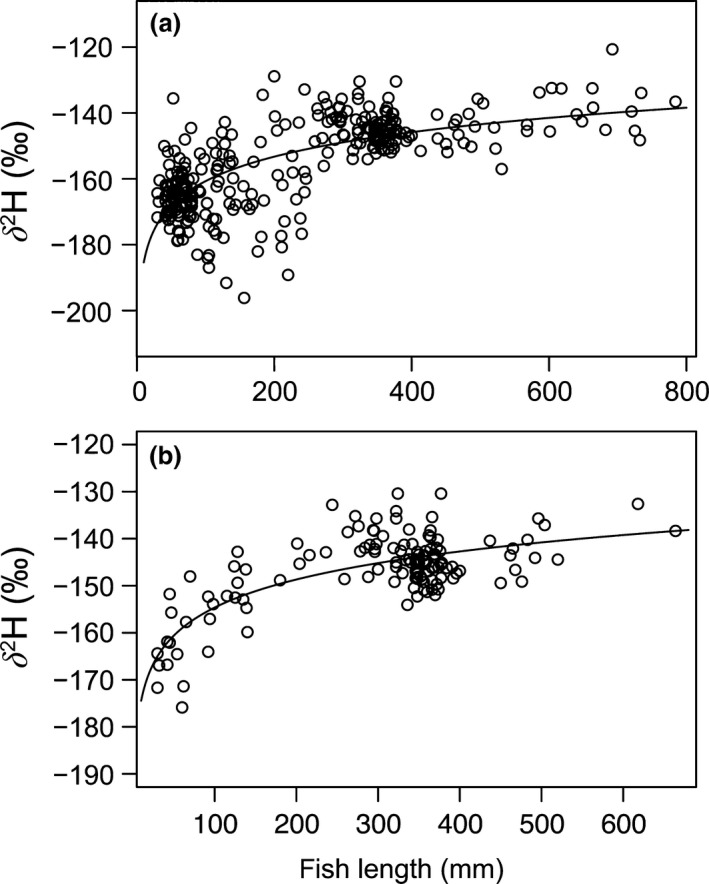
Relationship between size and δ^2^H values for (a) all fish (*n *=* *301) and (b) walleye (*n *=* *130) from all size classes (up to 800 mm) sampled in the south basin of Lake Winnipeg during 2008–2010 (note: no significant differences in water isotopes for those years; see 3)

A large range of fish size classes was collected in the period 2009–2010 in the south basin of Lake Winnipeg. We pooled all fish δ^2^H data from the south basin collected during the period 2008–2010 to assess the relationship with fork length. Our results showed a strong logarithmic correlation between fork length and δ^2^H values when the potential confounding variables (i.e., basin, time period) were considered a priori (adjusted R^2^ = .48, Figure 2a). This size relationship also held for walleye, which had the largest range in body sizes (adjusted R^2^ = .53, Figure 2b).

We found a fork‐length threshold of at least 250 mm was needed to clearly separate smaller fish with lower δ^2^H values from larger fish (Figure 2). Fish tissue δ^2^H values from the south basin were higher compared to those from the north basin for all time periods (2007 and 2008–2010, Figure 1), also along a longitudinal and latitudinal mixing gradient (Fig. S2).

**Figure 3:**
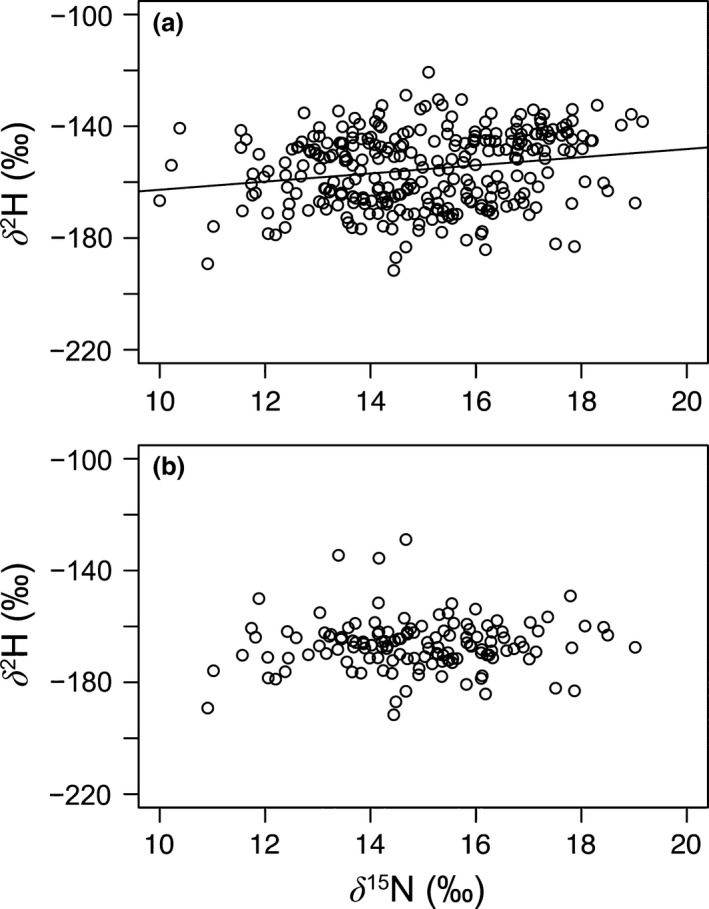
Trophic patterns of δ^2^H values in relation to δ^15^N for fish in the south basin of Lake Winnipeg from (a) the period 2008–2010. A subset of fish samples from (a) collected in 2008 and with sizes smaller than 250 mm is shown in (b). Linear regression is significant (*p *<* *.01) in (a) with a R^2^ of .04

Trophic associations were assessed using δ^15^N on the same subset of data previously used to examine fish size effects in the south basin (2008–2010), but no relationship was found with δ^2^H (adjusted R^2^ = .04, Figure 3a). However, each year of fish samples was comprised of a different range of fish size classes. The 2008 collection was the only year having individuals with sizes smaller than 250 mm. Therefore, a subset of δ^2^H data from Figure 3a collected in 2008 (i.e., size classes <250 mm) were related with δ^15^N to avoid potential confounding effects associated with size and, in this case, the correlation was even weaker (adjusted R^2 ^< .01, Figure 3b).

## 4 Discussion

Our results show the potential to use H isotopes as a means of tracing fish provenance and migration, providing they move between waters of sufficiently differing H isotope composition and the tissue used appropriately integrates and preserves the timing of migratory movements. Clearly, H isotope studies of animal movement in aquatic systems need to control for critical confounding effects associated with laboratory H isotope analyses and fish size. Another striking result from our study was the lack of evidence for a H isotope trophic effect, as revealed by corresponding δ^15^N measurements.

Our study supports experimental aquatic tests that suggested the so‐called trophic effect for δ^2^H is rather an apparent accumulation of δ^2^H with ambient waters across trophic levels that depends on the isotopic composition of the water and diet (Solomon et al., 2009; Soto et al., 2013c). The lack of any relationship between δ^15^N and δ^2^H in fish samples has been observed in other aquatic systems (Soto et al., 2011). The size correlation for fish δ^2^H values in Lake Winnipeg could be caused by the H isotopic compounding effect, whereby all prey and predator tissues reflect a fractional contribution from ambient waters, and higher trophic‐level consumers simply additively compound this effect. For tracing food web links in aquatic systems, it is important to measure water δ^2^H values (temporally) and preferably all potential dietary end‐member δ^2^H values. In our case, we did not sample the potential dietary items extensively and we cannot therefore confidently apply mixing models to our data. Nonetheless, as a proof of concept, we can use the mass‐balance model for fish tissue δ^2^H developed in Soto et al. (2013c) and assume no effect from metabolic water and a contribution of environmental water on protein isotope values (*p*_w_) of 33 % in fish. We measured the hydrogen isotopic composition of water in the south basin, which is −83‰, on average, for the time period 2008–2010. For that basin and period, if we speculate the smaller fish in our study (<250 mm, δ^2^H = −164‰ ± 11‰) are the predominant diet for larger fish that we collected (>400 mm), the mean modelled δ^2^H value of the latter should be ca. −137‰. The measured δ^2^H results of those large fish were −142‰ ± 7‰, which correspond well with the expectation of the fish isotope model when the contribution of environmental water is considered.

Alternatively, a second hypothesis related to metabolic water can also explain the pattern. Metabolic effects contribute to the body water δ^2^H pool increasing the relative contribution of metabolic products from diet but decreasing that of environmental water (Soto et al., 2013b). In natural systems, we would expect that H isotope contributions to fish tissue are a mixture of ambient water and metabolism. In our study, we suggest that smaller fish had different tissue δ^2^H values solely due to their higher growth rates as specific growth (or metabolic) rate usually declines with increasing fish size (Elliott & Hurley, 1995). This means a higher contribution of dietary lipids (with more negative δ^2^H values) and a relatively lower contribution from ambient water likely occurred in the smaller fish. This decrease in the relative contribution of ambient water to tissue H with increasing metabolic rates was also found experimentally for terrestrial bird tissues in relation to their drinking water (Storm‐Suke, Wassenaar, Nol, & Norris, 2012). Other studies have indicated that algal lipid δ^2^H values also showed changes associated with growth rate (Sachse et al., 2012).

For field studies, understanding the distinction between the compounding effect and a metabolic effect will be difficult, and so, we suggest working with fish and potentially other organisms that are of similar mass for any study involving δ^2^H measurements for the purpose of inferring movements or characteristics of ambient waters or lipid metabolism. Specifically, when applying hydrogen isotope analyses to ecological management issues (e.g., fisheries), researchers should carefully evaluate the size effects on tissue δ^2^H for the species of interest to account for any ontogenetic trophic and metabolic changes to then focus on specific size classes. If preliminary direct observations of potential size isotope patterns are unachievable, we advise selecting a priori a size range with the most limited extent possible for all fish species before any δ^2^H measurements.

Hydrogen isotope measurements appear to be a powerful tool to help determine the origin of fish with applications related to marine–freshwater fish movement studies (Whitledge et al., 2006) and potentially for use as a tracer for the authenticity of fish food products (Carter, Tinggi, Yang, & Fry, 2015) once relevant confounding effects are controlled (this study). We emphasize there is a need to control for H isotope exchange (Sauer, Schimmelmann, Sessions, & Topalov, 2009; Wassenaar et al., 2015) as well as the removal of lipids in animal tissues when measuring and interpreting H isotope data (Soto et al., 2013b; Wassenaar & Hobson, 2000). In our case, we were able to account for the water isotope contribution and metabolic effects when assessing the tissue δ^2^H values of fish smaller than 250 mm. Because water δ^2^H values in smaller water bodies may change seasonally over time, depending on the water residence time, it is important to measure water δ^2^H values over an appropriate temporal period when evaluating the utility of stable H isotopes for tracing fish provenance in freshwater aquatic systems.

Hydrogen isotopes are a relative newcomer to the toolbox for multiple stable isotope tracer approaches to understanding aquatic dynamics. However, unlike C and N stable isotope measurements, applying δ^2^H measurements effectively requires additional analytical care and attention.

## Conflict of Interest

None declared.

## Data Accessibility

Data used in the manuscript are present in the manuscript and its supporting information.
