# Supplementary material for: Using hydrogen isotopes of freshwater fish tissue as a tracer of provenance
Source: Ecol Evol. 2016 Oct 5;6(21):7776–82. doi: 10.1002/ece3.2519 (PMC6093159; doi:10.1002/ece3.2519)

Supplementary material

Table S1. Fork lengths (FL, mm) and *δ*^2^H values (‰) of fishes collected from Lake Winnipeg in 2007 and in the period 2008-2010. For descriptive purposes only, in this supplementary table, the lake was divided in North and South basins and the Narrows was included in those two lake sections using a threshold at latitude 51.7° N.

|  |  |  |  | *δ*^2^H | FL | |
| --- | --- | --- | --- | --- | --- | --- |
| Period | Species | N | Basin | Mean ± SD | Mean ± SD | Range |
| 2007 | Cisco | 11 | North | −171 ± 9 | 77 ± 25 | [45, 114] |
|  |  | 22 | South | −168 ± 10 | 99 ± 46 | [55, 235] |
|  | Emerald Shiner | 14 | North | −177 ± 11 | 63 ± 22 | [34, 93] |
|  |  | 28 | South | −176 ± 9 | 61 ± 20 | [25, 90] |
|  | Goldeye | 9 | South | −160 ± 12 | 110 ± 33 | [76, 165] |
|  | Rainbow smelt | 16 | North | −182 ± 13 | 73 ± 32 | [28, 130] |
|  |  | 12 | South | −164 ± 6 | 56 ± 9 | [40, 70] |
|  | Nine-spine stickleback | 3 | North | −187 ± 5 | 47 ± 3 | [45, 50] |
|  | Trout-perch | 7 | North | −161 ± 3 | 78 ± 32 | [30, 122] |
|  |  | 7 | South | −161 ± 5 | 50 ± 18 | [28, 80] |
|  | Walleye | 8 | North | −168 ± 13 | 115 ± 73 | [35, 250] |
|  |  | 24 | South | −164 ± 7 | 108 ± 49 | [40, 220] |
|  | White bass | 3 | North | −167 ± 3 | 48 ± 10 | [40, 60] |
|  |  | 17 | South | −164 ± 7 | 66 ± 34 | [20, 160] |
|  | Yellow perch | 7 | North | −173 ± 8 | 51 ± 25 | [31, 92] |
|  |  | 8 | South | −170 ± 6 | 59 ± 28 | [30, 111] |
|  |  |  |  |  |  |  |
| 2008-2010 | Cisco | 31 | North | −170 ± 12 | 162 ± 75 | [65, 392] |
|  |  | 80 | South | −164 ± 11 | 139 ± 82 | [44, 413] |
|  | Emerald Shiner | 46 | North | −170 ± 7 | 63 ± 16 | [30, 86] |
|  |  | 56 | South | −168 ± 8 | 61 ± 13 | [33, 85] |
|  | Goldeye | 22 | South | −172 ± 9 | 109 ± 42 | [50, 181] |
|  | Northern pike | 19 | South | −141 ± 8 | 599 ± 163 | [79, 784] |
|  | Rainbow smelt | 68 | North | −168 ± 10 | 82 ± 25 | [42, 150] |
|  |  | 4 | South | −162 ± 6 | 68 ± 32 | [47, 115] |
|  | Nine-spine stickleback | 10 | North | −165 ± 7 | 46 ± 6 | [33, 52] |
|  |  | 2 | South | −154 ± 6 | 45 ± 7 | [40, 50] |
|  | Trout-perch | 8 | North | −162 ± 8 | 68 ± 16 | [44, 83] |
|  |  | 27 | South | −159 ± 7 | 62 ± 16 | [36, 90] |
|  | Walleye | 13 | North | −173 ± 7 | 328 ± 163 | [52, 502] |
|  |  | 133 | South | −147 ± 8 | 296 ± 132 | [30, 664] |
|  | White bass | 24 | South | −165 ± 6 | 57 ± 31 | [35, 170] |
|  | White sucker | 12 | North | −174 ± 6 | 357 ± 74 | [220, 470] |
|  |  | 8 | South | −151 ± 4 | 369 ± 101 | [272, 531] |
|  | Yellow perch | 11 | North | −174 ± 20 | 59 ± 25 | [30, 125] |
|  |  | 13 | South | −168 ± 2 | 46 ± 11 | [30, 65] |

Figure S1. Relationships between spatial variables (latitude and longitude) and *δ*^2^H values for water samples collected in 2007 (a, c) and those collected in the period 2008-2010 (b, d).


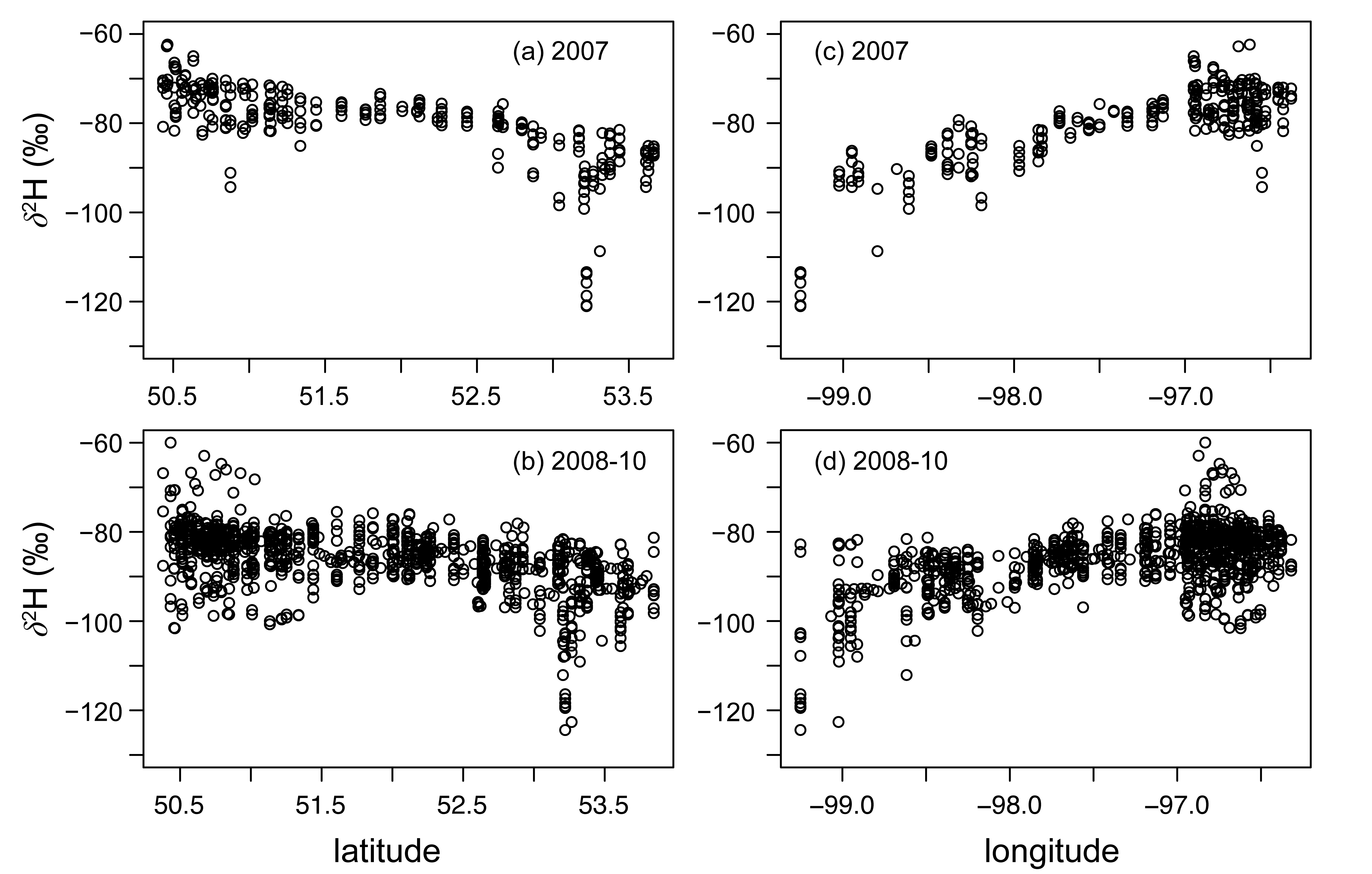


Figure S2. Relationships between spatial variables (latitude and longitude) and *δ*^2^H values for fish individuals smaller than 250 mm collected in 2007 (a, c) and those collected in the period 2008-2010 (b, d).


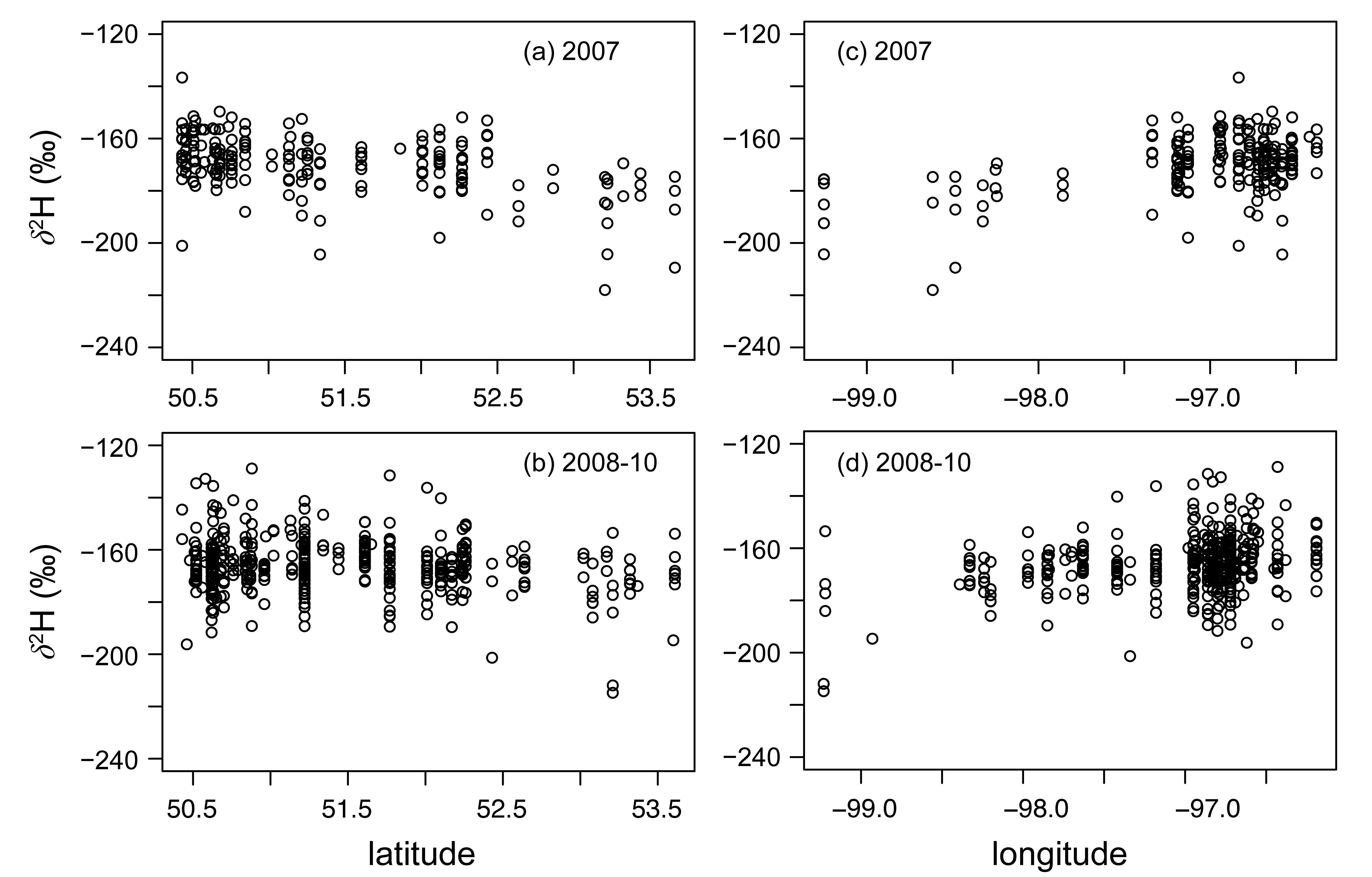

Supplement: Supplementary file 1 [file ECE3-6-7776-s001.docx]
